# Unintentional Pediatric Cannabis Exposures After Legalization of Recreational Cannabis in Canada

**DOI:** 10.1001/jamanetworkopen.2021.42521

**Published:** 2022-01-07

**Authors:** Daniel T. Myran, Nathan Cantor, Yaron Finkelstein, Michael Pugliese, Astrid Guttmann, Rebecca Jesseman, Peter Tanuseputro

**Affiliations:** 1 Clinical Epidemiology Program, Ottawa Hospital Research Institute, Ottawa, Ontario, Canada; 2 Department of Pediatrics, University of Toronto, Toronto, Ontario, Canada; 3 ICES (formerly the Institute for Clinical Evaluative Sciences), Ontario, Canada; 4 Canadian Centre on Substance Use and Addiction, Ottawa, Ontario, Canada; 5 Department of Medicine, Ottawa Hospital Research Institute, University of Ottawa, Ottawa, Ontario, Canada

## Abstract

This cross-sectional study examines changes in emergency department visits and hospitalizations due to cannabis exposures among children after legalization of recreational cannabis in Canada.

## Introduction

Previous studies have documented increases in cannabis exposures among young children after legalization of recreational cannabis.^1,2,3^ Increasing evidence has implicated commercially produced edible cannabis products as a key factor associated with these increases.^3^ Canada took a 2-phased approach to legalizing recreational cannabis. Initially, the sale of cannabis flower, seeds, and oils was permitted, and after 1 year, this expanded to a wider variety of products, including cannabis edibles.^4^ We evaluated changes in pediatric emergency department (ED) visits and hospitalizations due to cannabis exposures associated with these changes.

## Methods

This repeated cross-sectional study was authorized under section 45 of Ontario’s Personal Health Information Protection Act and approved by the privacy and legal office of ICES (formerly the Institute for Clinical Evaluative Sciences). Section 45 allows ICES to collect personal health information without consent for the purpose of health system evaluation and improvement. We followed the Strengthening the Reporting of Observational Studies in Epidemiology (STROBE) reporting guideline for cross-sectional studies.

We identified all ED visits and related hospitalizations due to cannabis exposures among 2.35 million children aged 0 to 9 years in Ontario, Canada, between January 1, 2016, and March 31, 2021. We compared trends and characteristics of ED visits over 3 periods: prelegalization (January 2016-September 2018); the period after legalization of flower products, or period 1 (October 2018-January 2020); and the period after commercial edibles became available, or period 2 (February 2020-March 2021). Poisson models were used to calculate incidence rate ratios (IRRs) for change in monthly rates of visits. Health administrative data sets were linked using encoded identifiers and analyzed at ICES (eMethods in the Supplement). All tests of significance were 2-sided, and *P* values < .05 were considered statistically significant. Data analysis was conducted from June through August 2021 using Stata statistical software version 17.0 (StataCorp).

## Results

There were 522 ED visits due to cannabis exposures among children (mean [SD] age, 3.8 [2.6] years; 281 visits [53.8%] among boys) including 81 visits during prelegalization, 124 visits during period 1, and 317 visits during period 2. The proportion of cannabis-related ED visits with hospitalization increased significantly after the introduction of edibles (122 visits [38.5%] during period 2 vs 29 visits [23.4%] during period 1 and 20 visits [24.7%] during the prelegalization period; *P* = .002). There were 19 ED visits (3.6%) with intensive care unit admission; no deaths were recorded (Table).

**Table.** Cannabis Exposures Among Children by Time Period

	Prelegalization^a^	Period 1^b^	Period 2^c^	*P* value^d^
**Cannabis exposure ED visits by characteristic**				
Total visits, No. (monthly mean)	81 (2.5)	124 (7.8)	317 (22.6)	NA
Age, mean (SD)	3.6 (2.8)	3.5 (2.8)	4.0 (2.5)	.18
Sex, No. (%)				
Boys	44 (54.3)	78 (62.9)	159 (50.2)	.054
Girls	37 (45.7)	46 (37.1)	158 (49.8)	
Hospitalized, No. (%)	20 (24.7)	29 (23.4)	122 (38.5)	.002
**Cannabis ED exposure visits per 100 000 population members**				
Monthly rate, mean (95% CI)	0.16 (0.11-0.21)	0.51 (0.43-0.59)	1.48 (1.30-1.66)	NA
Annualized rate	1.96	6.14	17.75	NA
IRR (95% CI)				
Unadjusted	1 [Reference]	3.14 (2.37-4.16)	9.12 (7.15-11.65)	Period 1: < .001
				Period 2: < .001
Adjusted for monthly time trend^e^	1 [Reference]	1.33 (0.85-2.10)	2.23 (1.17-4.27)	Period 1: .21
				Period 2: .01
**Cannabis ED exposure visits per 1000 all-cause poisoning ED visits^f^**				
Monthly rate, mean (95% CI)	6.84 (4.80-8.88)	28.85 (22.07-35.63)	95.03 (80.54-109.52)	NA
IRR (95% CI)				
Unadjusted	1 [Reference]	3.81 (2.88-5.04)	13.05 (10.22-16.66)	Period 1: .001
				Period 2: .001
Adjusted for monthly time trend^g^	1 [Reference]	1.50 (0.95-2.38)	2.87 (1.49-5.52)	Period 1: .08
				Period 2: .002

Abbreviations: ED, emergency department; IRR, incident rate ratio; NA, not applicable.

^a^ 33 months: January 2016-September 2018.

^b^ Legalization of flower-based cannabis products, 16 months: October 2018-January 2020.

^c^ Introduction of legal commercial edible cannabis products, 14 months: February 2020-March 2021.

^d^ Periods 1 and 2 are compared with the prelegalization period.

^e^ IRR for monthly time trend: 1.04 (95% CI, 1.02-1.05; *P* < .001).

^f^ ED visits related to all pharmaceutical and nonpharmaceutical poisonings.

^g^ IRR for monthly time trend: 1.04 (95% CI, 1.02-1.06; *P* < .001).

Rates of ED visits associated with cannabis exposures increased from January 2016 to March 2021 (Figure). Period 1 (IRR, 3.13; 95% CI, 2.37-4.16; *P* < .001) and period 2 (IRR, 9.12; 95% CI, 7.15-11.65; *P* < .001) were associated with increases in visits compared with the prelegalization period, with a larger IRR for period 2. After adjusting for an increasing time trend in ED visits due to cannabis exposures throughout the study period, period 2 continued to be associated with an increase in visits (IRR, 2.23; 95% CI, 1.17-4.27; *P* = .01) (Table). Period 2 overlapped with the COVID-19 pandemic. During this time, pediatric ED visits due to cannabis exposures increased despite a decrease in total poisoning-related pediatric ED visits; the mean (SD) monthly count of visits was 312.3 (102.3) visits in the year prior to the pandemic vs 263.5 (100.4) visits during the first year of the pandemic.

**Figure.**
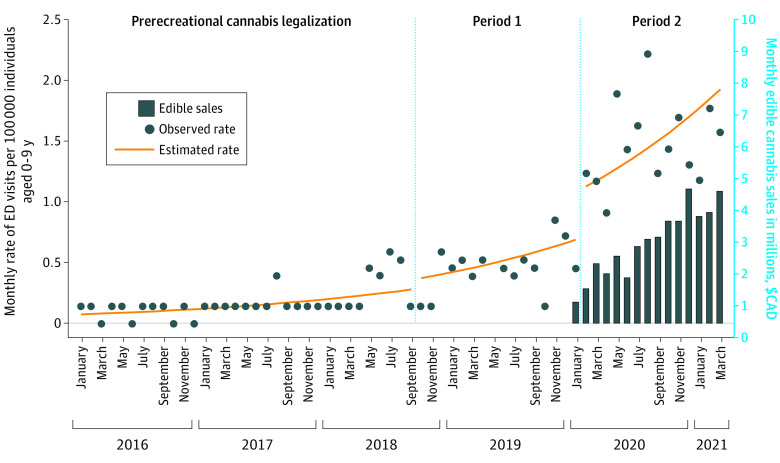
Monthly Emergency Department (ED) Visits Due to Cannabis Exposures Among Children Blue dots indicate observed rate; orange line, time-adjusted estimated rate; blue bars, monthly value of sales of legal commercial cannabis edible products (in millions $CAD); period 1, legalization of flower-based cannabis products; period 2, introduction of legal commercial edible cannabis products. To comply with privacy requirements at ICES (formerly the Institute for Clinical Evaluative Sciences), the rate of visits in months with 1 to 5 ED visits has been adjusted to represent the mean rate (0.14 monthly visits per 100 000 individuals) of all months during the study with 1 to 5 ED visits. The estimated trend line and analysis are based on unadjusted observed data.

## Discussion

This repeated cross-sectional study found significant increases in the frequency and severity of ED visits due to cannabis exposures among children after the legalization of recreational cannabis. These findings suggest that the introduction of legal commercial edible cannabis products was a key factor associated with changes in ED visit frequency and severity. Rates of pediatric cannabis ED exposures found in this study were 7-fold higher than rates reported in Colorado after recreational cannabis legalization.^1^ These population-level findings suggest that prior work from single centers may have underestimated the burden associated with pediatric cannabis exposures. Increases in ED visit frequency and severity occurred despite strict regulations that largely exceed US regulations (eg, a maximum of 10 mg of tetrahydrocannabinol per entire edible package, child-resistant packaging, and marketing restrictions) and consumer education campaigns.^5^

Our study was limited by lack of data on the source and type of cannabis ingested, and it is possible that cannabis from illicit sources and nonedible products contributed to the increase in visits. The legal cannabis retail market in Ontario has expanded rapidly since the start of period 2, and the number of legal cannabis stores is expected to increase 3-fold in the coming years.^6^ Further regulatory measures, such as limiting formulations and appearance of commercial edibles, combined with education for parents and caregivers, may be required to decrease pediatric cannabis exposures.

## Notes

Supplement.
eMethods.

eReferences.
