# Supplementary material for: Unintentional Pediatric Cannabis Exposures After Legalization of Recreational Cannabis in Canada
Source: JAMA Netw Open. 2022 Jan 7;5(1):e2142521. doi: 10.1001/jamanetworkopen.2021.42521 (PMC8742190; doi:10.1001/jamanetworkopen.2021.42521)
Supplement: Supplement. — eMethods. eReferences. [file jamanetwopen-e2142521-s001.pdf]

Supplemental Online Content

Myran DT, Cantor N, Finkelstein Y, et al. Unintentional pediatric cannabis exposures after legalization of recreational cannabis in Canada. *JAMA Netw Open*. 2022:5(1):e2142521. doi:10.1001/jamanetworkopen.2021.42521

eMethods.
eReferences.

This supplemental material has been provided by the authors to give readers additional information about their work.

© 2022 Myran DT et al. *JAMA Network Open.*

eMethods.

This supplemental material has been provided by the authors to give readers additional information about their work.

Case Definitions

The primary outcomes in this study were ED visits due to pediatric cannabis exposures. ED visits due to cannabis exposures were defined as visits where one of the following ICD-10 codes was listed as the main or contributing reason for visit: T40.7 (Poisonings by cannabis, including derivatives); and F12.X (Mental and behavioral disorders due to use of cannabinoids). We examined the clinical outcomes of ED visits as the proportion of visits resulting in hospitalization and/or admission to the intensive care unit (ICU).

A cannabis-related code (T40.7 or F12.X) was listed as the main (most responsible) code for the visit in 96.4% (n=503) of ED visits. For seven visits (1.3% of total visits), poisoning by a substance other than cannabis was listed as the most responsible diagnosis and the cannabis code was listed as a contributing reason to the visit. The most frequently-coded cause of visit (n=470, 90%) was "poisoning by cannabis or derivatives" (ICD-10 code T40.7). The second most frequently-coded cause of visit was "acute intoxication due to use of cannabinoids" (ICD-10 code F12.0), which occurred for 7.1% (n=37) of visits.

To contextualize our results due to changes in health seeking behavior during the COVID-19 pandemic (e.g. reduced all-cause ED visits)(1) and public health measures (e.g. school and daycare closures that led to children spending more time at home) we compared changes in ED

© 2022 Myran DT et al. *JAMA Network Open.*

visits due to cannabis exposures to ED visits due to pharmaceuticals poisonings (ICD-10 Codes T36-T50) and non-pharmaceutical poisonings (ICD-10 Codes T51-T65) in children aged 0-9 years.

© 2022 Myran DT et al. *JAMA Network Open.*

Data Sources

Data were obtained using linked health administrative data held by ICES, an independent, non-profit research institute whose legal status under Ontario's health information privacy law allows it to collect and analyze health care and demographic data, without consent, for health system evaluation and improvement. Data on child characteristics and health care utilization were obtained through the following linked, de-identified health administrative databases at ICES: 1) the Registered Persons Database which captures demographic information including age and sex; 2) the National Ambulatory Care Reporting System, which captures all ED visits within Ontario; 3) the Discharge Abstract Database, which includes clinical and demographic data on all hospitalizations in Ontario. These datasets were linked using unique encoded identifiers and analyzed at ICES. This project was authorized under section 45 of Ontario's Personal Health Information Protection Act thus not requiring review by a Research Ethics Board.

We obtained the monthly value (in $CAD) of all commercial edible cannabis sold in Ontario from the Ontario Cannabis Store (OCS).(2) The OCS has reported the breakdown of sales by cannabis products (i.e., edibles, flower, etc.) from all legal retail stores in Ontario and through its own online website since the beginning of period 2 (sale of legal commercial edible cannabis products). We considered the period 2 to start in February 2020 - the first full month of exposures to legal commercial edible products (products became available in late January 2020).

© 2022 Myran DT et al. *JAMA Network Open.*

Analyses

We present descriptive statistics including means, frequencies, and proportions to characterize and compare ED visits due to pediatric cannabis exposures across the three regulatory time periods. We tested for significant differences between ED visit characteristics at all three time periods through Chi-Squared tests for categorical variables and one-way ANOVA for continuous variables. We calculated the mean monthly rate of ED visits with 95% confidence intervals (CIs) for each period. We calculated incidence rate ratios (IRRs) with 95% confidence intervals through generalized linear models using a Poisson distribution to assess the separate impacts of each regulatory period. Our unit of analysis was the monthly count of ED visits due to cannabis. First, we estimated the increase using a model with a three-level categorical variable to represent the distinct regulatory periods (0 = pre-legalization, 1 = *Period 1,* 2 = *Period 2*). We ran a separate model which also included a linear covariate for time (in months) to control for time trends. We offset our models by the logarithmic-transformed total population of Ontario aged 0 to 9 years at-risk (alive and eligible for universal health insurance each month) to examine changes in visits per capita. To examine potential COVID-19 effects, we ran the same models with the offset as the logarithmic-transformed total poisonings (including those related to cannabis) to examine changes in visits as a proportion of overall poisoning visits. Inclusion of an adjustment factor in our models for over-dispersion did not change model fit or variance estimation and therefore we did not include the adjustment factor in final models. Durbin-Watson tests indicated no requirement to adjust for autocorrelation. Data analysis was conducted between June 2021 and August 2021 using STATA Version 17.0 (College Station,

© 2022 Myran DT et al. *JAMA Network Open.*

TX). All tests of significance were two-sided and *P* values <.05 were considered statistically significant.

© 2022 Myran DT et al. *JAMA Network Open.*

eReferences.

1. Finkelstein Y, Maguire B, Zemek R, Osmanlliu E, Kam AJ, Dixon A, et al. Effect of the COVID-19 Pandemic on Patient Volumes, Acuity, and Outcomes in Pediatric Emergency Departments: A Nationwide Study. Pediatr Emerg Care. 2021 Jun;37(8):427.
2. Ontario Cannabis Store. A YEAR IN REVIEW (April 1, 2020 - March 31 2021). 2021.

© 2022 Myran DT et al. *JAMA Network Open.*
